# Supplementary material for: Modeling cystic fibrosis patient prognosis: Nomograms to predict lung transplantation and survival prior to highly effective modular therapy
Source: PLoS One. 2024 Dec 5;19(12):e0292568. doi: 10.1371/journal.pone.0292568 (PMC11620394; doi:10.1371/journal.pone.0292568)
Supplement: Attachment — Submitted filename: Response to Reviewers .docx [file pone.0292568.s001.docx]

Journal requirements:

When submitting your revision, we need you to address these additional requirements.

1. Please ensure that your manuscript meets PLOS ONE's style requirements, including those for file naming. The PLOS ONE style templates can be found at

[https://journals.plos.org/plosone/s/file?id=wjVg/PLOSOne_formatting_sample_main_body.pdf](https://urldefense.com/v3/__https:/journals.plos.org/plosone/s/file?id=wjVg*PLOSOne_formatting_sample_main_body.pdf__;Lw!!BZ50a36bapWJ!uQ4kAL3oWT7Ewnzp0yEocSv8pKr7ekeASsvtfyeJeL6Mz0hnppQMujcAEUP9DKYdxCttNSWMcz3JJESMPC6HqA$) and

[https://journals.plos.org/plosone/s/file?id=ba62/PLOSOne_formatting_sample_title_authors_affiliations.pdf](https://urldefense.com/v3/__https:/journals.plos.org/plosone/s/file?id=ba62*PLOSOne_formatting_sample_title_authors_affiliations.pdf__;Lw!!BZ50a36bapWJ!uQ4kAL3oWT7Ewnzp0yEocSv8pKr7ekeASsvtfyeJeL6Mz0hnppQMujcAEUP9DKYdxCttNSWMcz3JJEQMmx9g0A$)

2. We noticed you have some minor occurrence of overlapping text with the following previous publication(s), which needs to be addressed:

Culture independent markers of nontuberculous mycobacterial (NTM) lung infection and disease in the cystic fibrosis airway - [https://doi.org/10.1016/j.tube.2022.102276](https://urldefense.com/v3/__https:/doi.org/10.1016/j.tube.2022.102276__;!!BZ50a36bapWJ!uQ4kAL3oWT7Ewnzp0yEocSv8pKr7ekeASsvtfyeJeL6Mz0hnppQMujcAEUP9DKYdxCttNSWMcz3JJEQlIL8Aiw$)

*Thank you for identifying this overlapping text. We have extensively rewritten this introductory paragraph to insure it is completely original, and to improve the clarity of the text.*

In your revision ensure you cite all your sources (including your own works), and quote or rephrase any duplicated text outside the methods section. Further consideration is dependent on these concerns being addressed.

3. Please note that PLOS ONE has specific guidelines on code sharing for submissions in which author-generated code underpins the findings in the manuscript. In these cases, all author-generated code must be made available without restrictions upon publication of the work. Please review our guidelines at [https://journals.plos.org/plosone/s/materials-and-software-sharing#loc-sharing-code](https://urldefense.com/v3/__https:/journals.plos.org/plosone/s/materials-and-software-sharing*loc-sharing-code__;Iw!!BZ50a36bapWJ!uQ4kAL3oWT7Ewnzp0yEocSv8pKr7ekeASsvtfyeJeL6Mz0hnppQMujcAEUP9DKYdxCttNSWMcz3JJEQZkBPQvA$) and ensure that your code is shared in a way that follows best practice and facilitates reproducibility and reuse.

4. We note that you have indicated that there are restrictions to data sharing for this study. For studies involving human research participant data or other sensitive data, we encourage authors to share de-identified or anonymized data. However, when data cannot be publicly shared for ethical reasons, we allow authors to make their data sets available upon request. For information on unacceptable data access restrictions, please see [http://journals.plos.org/plosone/s/data-availability#loc-unacceptable-data-access-restrictions](https://urldefense.com/v3/__http:/journals.plos.org/plosone/s/data-availability*loc-unacceptable-data-access-restrictions__;Iw!!BZ50a36bapWJ!uQ4kAL3oWT7Ewnzp0yEocSv8pKr7ekeASsvtfyeJeL6Mz0hnppQMujcAEUP9DKYdxCttNSWMcz3JJESMZLEabA$).

Before we proceed with your manuscript, please address the following prompts:

a) If there are ethical or legal restrictions on sharing a de-identified data set, please explain them in detail (e.g., data contain potentially identifying or sensitive patient information, data are owned by a third-party organization, etc.) and who has imposed them (e.g., a Research Ethics Committee or Institutional Review Board, etc.). Please also provide contact information for a data access committee, ethics committee, or other institutional body to which data requests may be sent.

*Cystic Fibrosis Foundation Patient Registry (CFFPR) data were used for this study. The Cystic Fibrosis Foundation provides CFFPR data upon request, thus, it is not generally publicly available. The public can apply for CFFPR via this website:* [*https://www.cff.org/researchers/patient-registry-data-requests*](https://www.cff.org/researchers/patient-registry-data-requests) *and so others can access the data in the same manner we, the authors, obtained them. This is how we were approved to use the specific data used in the study, which was provided by the CFF directly.*

b) If there are no restrictions, please upload the minimal anonymized data set necessary to replicate your study findings to a stable, public repository and provide us with the relevant URLs, DOIs, or accession numbers. Please see [http://www.bmj.com/content/340/bmj.c181.long](https://urldefense.com/v3/__http:/www.bmj.com/content/340/bmj.c181.long__;!!BZ50a36bapWJ!uQ4kAL3oWT7Ewnzp0yEocSv8pKr7ekeASsvtfyeJeL6Mz0hnppQMujcAEUP9DKYdxCttNSWMcz3JJESZE7jvYA$) for guidelines on how to de-identify and prepare clinical data for publication. For a list of recommended repositories, please see [https://journals.plos.org/plosone/s/recommended-repositories](https://urldefense.com/v3/__https:/journals.plos.org/plosone/s/recommended-repositories__;!!BZ50a36bapWJ!uQ4kAL3oWT7Ewnzp0yEocSv8pKr7ekeASsvtfyeJeL6Mz0hnppQMujcAEUP9DKYdxCttNSWMcz3JJESsH-pGQQ$). You also have the option of uploading the data as Supporting Information files, but we would recommend depositing data directly to a data repository if possible.

Please update your Data Availability statement in the submission form accordingly.

5. Please upload a new copy of Figures 1 and 2 as the detail were not clear. Please follow the link for more information: [https://blogs.plos.org/plos/2019/06/looking-good-tips-for-creating-your-plos-figures-graphics/"](https://urldefense.com/v3/__https:/blogs.plos.org/plos/2019/06/looking-good-tips-for-creating-your-plos-figures-graphics/*22__;JQ!!BZ50a36bapWJ!uQ4kAL3oWT7Ewnzp0yEocSv8pKr7ekeASsvtfyeJeL6Mz0hnppQMujcAEUP9DKYdxCttNSWMcz3JJERLq0Et3Q$) [https://blogs.plos.org/plos/2019/06/looking-good-tips-for-creating-your-plos-figures-graphics/](https://urldefense.com/v3/__https:/blogs.plos.org/plos/2019/06/looking-good-tips-for-creating-your-plos-figures-graphics/__;!!BZ50a36bapWJ!uQ4kAL3oWT7Ewnzp0yEocSv8pKr7ekeASsvtfyeJeL6Mz0hnppQMujcAEUP9DKYdxCttNSWMcz3JJET8EyQdfw$)

[Note: HTML markup is below. Please do not edit.]

Reviewers' comments:

Reviewer's Responses to Questions

**Comments to the Author**

1. Is the manuscript technically sound, and do the data support the conclusions?

The manuscript must describe a technically sound piece of scientific research with data that supports the conclusions. Experiments must have been conducted rigorously, with appropriate controls, replication, and sample sizes. The conclusions must be drawn appropriately based on the data presented.

Reviewer #1: Partly

Reviewer #2: No

2. Has the statistical analysis been performed appropriately and rigorously?

Reviewer #1: No

Reviewer #2: No

3. Have the authors made all data underlying the findings in their manuscript fully available?

The [PLOS Data policy](https://urldefense.com/v3/__http:/www.plosone.org/static/policies.action*sharing__;Iw!!BZ50a36bapWJ!uQ4kAL3oWT7Ewnzp0yEocSv8pKr7ekeASsvtfyeJeL6Mz0hnppQMujcAEUP9DKYdxCttNSWMcz3JJETC85XrqA$) requires authors to make all data underlying the findings described in their manuscript fully available without restriction, with rare exception (please refer to the Data Availability Statement in the manuscript PDF file). The data should be provided as part of the manuscript or its supporting information, or deposited to a public repository. For example, in addition to summary statistics, the data points behind means, medians and variance measures should be available. If there are restrictions on publicly sharing data—e.g. participant privacy or use of data from a third party—those must be specified.

Reviewer #1: No

Reviewer #2: Yes

4. Is the manuscript presented in an intelligible fashion and written in standard English?

PLOS ONE does not copyedit accepted manuscripts, so the language in submitted articles must be clear, correct, and unambiguous. Any typographical or grammatical errors should be corrected at revision, so please note any specific errors here.

Reviewer #1: Yes

Reviewer #2: Yes

5. Review Comments to the Author

Please use the space provided to explain your answers to the questions above. You may also include additional comments for the author, including concerns about dual publication, research ethics, or publication ethics. (Please upload your review as an attachment if it exceeds 20,000 characters)

Reviewer #1: Dear authors,
1. the subset of data you chosen include people with CF until their 40, limited to 2014. Age is "age at follow-up" or "age at registry entry"?

*We agree this information is important and we have made edits to further describe age in the Patients section of the Materials and Methods section.*

*Age is defined as age at follow-up. Age at follow-up is calculated as the difference between review year and date of birth year. Review year is the year when the clinical visit(s) occurred, so age is their age in that year. For patients that died or had a lung transplant, we calculated age at least one year prior to the event. We did this to enable additional time prior to the event of interest. Additionally, we assumed if a patient had a lung transplant or died, it would be beneficial for them to be listed for lung transplant earlier rather than later. For patients that were censored due to the end of the study period or lost to follow-up, we calculated their age as age in the most recently observed year.*

2. Despite the choice of limiting age at 40 could be likely based on the age at death extrapolated in the paper of Schluchter et al, censoring at 2014 can be seen as a major limitation, considering the rapid changing clinical landscape of the disease, regardless HEMT. Also, many trials on HEMT were already ongoing during 2014. How these predictions could be useful now?

*We agree that the CF treatment landscape has evolved rapidly over the past two decades. In 2014 approximately 6% of the U.S. CF population had access to HEMT. A number of trials were ongoing for different combinations of modulators; however, these formulations are now recognized as providing very small incremental benefit and are rarely used today. It wasn’t until mid-2020 (following approval of E/T/I in November 2019) that the majority of pwCF had access to HEMT. As noted in the last sentence of the Introduction, “The current study aims to demonstrate the validity of this method using data pre-HEMT, as a step towards a current prediction of differing time periods of risk based on patient-specific characteristics to provide a more detailed understanding of a specific patient’s prognosis.” In the discussion, we more thoroughly explain our reasoning for using this data specifically: “Finally, the current nomograms were developed using data that generally predated widespread use of highly effective CFTR modulator therapy (HEMT) in the United States CF population. CFTR modulators improve outcomes for a majority of individuals with CF [2, 3, 5] and may deliver a sustained benefit over time [47]. Clinical trials excluded patients with advanced lung disease, thus there is little available data to guide the degree of disease modification in the period included within this study. It is now apparent that most individuals receiving HEMT will experience slower progression towards transplant or death in this cohort, but current CFF-sponsored guidelines for lung transplant referral suggest that transplant referral not be delayed based on use of CFTR modulators [2, 3, 5, 47]. Going forward, these findings can serve as a comparison for future cohorts who are on HEMT.”*

*We acknowledge that these nomograms are not intended for clinical use. Extensive external validation would be required before introducing any tool into clinical care, and given the magnitude of the benefit from HEMT, it is certain that revisions are required. We do feel strongly that this method to determine probability and time to lung transplant or death is of use to build upon for the current CF population, and to model the need for transplant relative to historic trends and for those that do not benefit from HEMT. To the extent that a small number of pwCF receiving HEMT were included in this analysis only serves to make the analysis more relevant to the current population. We have reviewed the Discussion section and made edits to reinforce this point.*

3. Considering the specific care of the CF disease, even back to 2014, lung transplant should be conceived as a competitive event of death.

*We agree that lung transplant is a competitive event of death. That is why we defined the event as lung transplant or death, whichever came first. Reason for death was not well documented in the dataset and since lung disease is the cause of the death for the majority of CF patients, we assumed that if a CF patient died that indicated the patient needed a lung transplant .” (*[*https://www.cdc.gov/scienceambassador/documents/cystic-fibrosis-fact-sheet.pdf*](https://www.cdc.gov/scienceambassador/documents/cystic-fibrosis-fact-sheet.pdf)*). We added to the Methods section to further explain this.*

4. With regard to missingness, it is not clear why you worked only on the imputed dataset with lower standard error and not on the combined imputed datasets (how many, for instance?);

*We compared 25 different imputed datasets and the greatest differences among the standard errors were 0.000465 for FEV1 % predicted and 0.000000202 for BMI and the same for 10 decimal places for the other quantitative predictors, so although we chose the smallest standard error, the values were very close to each other or nearly identical.* *Similarly,* *the greatest differences among the means were 0.099111 for FEV1 % predicted, 0.008916 for BMI, 0.001396 for age of diagnosis, 0.000202 for minimum age of death/transplant and were identical for the other quantitative predictors. Thus, although we chose the smallest standard error, the standard error and mean values were very close to each other or nearly identical. We have incorporated details relating to the number of imputation conducted and our imputed dataset selection to the Methods section. We agree this is important information to include—thank you for bringing it to our attention.*

5. By consequence, the validation as well should have be done on all imputed datasets.

*As mentioned above in response to question 4, the differences between the standard errors and between the means of the quantitative predictors among the imputations were very small, so we chose not to repeat the analysis on all of the imputed datasets.*

*We chose to use leave one out cross-validation (LOOCV) to carry out internal validation of our logistic regression model and “bootstrapped resampling with 1000 repetitions was used to validate the Cox regression model with R function validate” (Model Validation section of the Methods section). Additionally, we built the models with a training dataset with a randomly selected dataset consisting of 90% of the observations and assessed the model accuracy with the remaining 10% of observations. The validation process is described in more detail in response to question 1 from reviewer 2 below and we have edited Methods section to explain this in more detail.*

6. Findings from the fitted model are however limited to an US audience only (e.g., variabile insurance not available in Europe)

*Lung transplant allocation is decided by lung allocation score (LAS) for 60% of the world; however, countries have country/region-specific allocation systems (Gottlieb, 2017). Thus, we felt it was important to address this issue in a country-specific fashion. Ramos et al. (2017) found Medicaid insurance had an estimated hazard ratio of 1.67 risk of death in a univariate model and an estimated hazard ratio of 1.16 risk of death in a multivariate model. Ramos et al. (2017) found this using a retrospective cohort of the Cystic Fibrosis Foundation Patient Registry as we did with our study, so we felt it was important to assess whether the same predictors could be predictive of lung transplant/death. DuBay et al. (2017) also assessed the “frequency and variation in Medicaid transplantation, and post-transplant survival in Medicaid patients” and determined “Medicaid organ transplant beneficiaries had significantly lower survival compared to Privately insured beneficiaries;” this further supports our decision to assess the impact of insurance type on probability and time to lung transplant/death.*

*We agree that these results are based on the population of CF patients in the United States who are attending CF Care Centers, as the model was created using data was derived from the US Cystic Fibrosis Foundation Patient Registry. There are any number of differences in health care delivery and accessibility between various countries that could impact these results. We have added a comment noting this to the discussion.*

7. Which time scale for the Cox Model?

*Thank you for your question; we agree this needs to be further emphasized in our manuscript.*

*The time scale for the Cox Model is age in years, specifically age until death or lung transplant, whichever came first or age until censored for those that did not die or did not have lung transplant. In the Materials and Methods section Predictors and Outcome variables, this is specifically mentioned: “For the Cox model, time to event was set up as the minimum of the age of lung transplant or death, with age in the most recent review year used for censored observations.” We have added to this sentence to more thoroughly explain the time scale, added details indicating this in the Cox Multiple Regression Model section of the Results, and added to the Table 3 description. As noted in Fig. 2. Nomogram for probability of lung transplant or death free survival for 2 years, the measurement scale is in years. Although the nomogram is based on the Cox model results, it is depicting probability of lung transplant or death free survival for 2 years, defining time in years in the current moment to 2 years in the future.*

Reviewer #2: This manuscript proposes a prediction model for lung transplantation and survival using standard logistic and Cox regression approaches. The authors utilized Cystic Fibrosis Foundation Patient Registry (CFFPR), which offers gigantic sample size with a small amount of missing data.

Major comments:
1. My major concern is the non-comparative approaches. Why authors did not try machine learning approaches such as lasso/random forest using rigorous cross-validations instead of a leave-one-out approach. Given this huge sample size, this is feasible. Here authors present prediction models, so I believe that it is essential to focus on prediction performances such as AUROC (sensitivity and specificity) rather than model goodness of fits. Correlations among predictors also need to be reported.

*Comparative approaches were used. With both the multiple logistic regression model and the Cox model, predictors were selected based on significance of predictors (p ≤ 0.05), Akaike information criterion (AIC), and concordance index, i.e., the predictive performance of different combinations of predictors was assessed and compared to select a best fitting models.*

*The major motivations for this paper were effective communication between the clinicians at CF Care Centers, lung transplant centers, and the patients and “a more personalized approach to transplant referral and listing” (Introduction of this manuscript). Thus, we used nomograms: “Nomograms are meant to personalize estimations of survival and potentially ease communication between the CF clinician and patient and the lung transplant care team about the characteristics of the CF patient affecting both their probability of lung transplant or death and their probability of lung-transplant-free survival” (Introduction of this manuscript). The R package rms with function nomogram was used to construct the nomograms in this study; The rms package “contains functions for binary logistic regression models” and “was especially written to work with … Cox regression models” (https://cran.r-project.org/package=rms). This was one of the reasons we chose to use multiple logistic regression and Cox regression rather than lasso or random forest.*

*Additionally with lasso, k-fold CV is conducted within the model selection whereas we carried out validation separately with leave-one-out cross-validation (LOOCV). With LOOCV, we were able to maximize the number of observations for training while still having independent validation observations for testing and training datasets that nearly identical.*

*Because we wanted to allow our models to be subject-specific, i.e., we wanted providers/patients to be able to use their exact clinical values to make predictions about their prognosis, random forest was not used. Random forest structures all predictors as categorical. As we noted in the discussion section of our manuscript, Nkam et al. (2017) already developed a nomogram using only categorical predictors using French registry data, which is not able to predict specific prognosis for specific patients.*

*The performance of the logistic regression model was assessed using the concordance index, which is equivalent to AUROC when the dependent variable is binary (Austin and Steyerberg, 2012). Additionally, the logistic regression was validated with LOOCV, which is not a goodness-of-fit measure. Since LOOCV and AUROC were not options for the Cox regression modeling, “bootstrapped resampling with 1000 repetitions was used to validate the Cox regression model with R function validate” (Model Validation section of the Methods section).* *Additionally, training and testing datasets consisting of random selections of 90% and 10% of the dataset, respectively, were used to build and internally validate the models, respectively. We have added to the Model Development section of the Methods section to describe these training and testing datasets.*

*Since there were ~20 variables there are at least 400 correlations between them. Thus, we chose not to report them all. However, we do agree they were of important consideration in our modeling and should be described. We agree this is important information so we added a description of the correlation assessment to the Predictors and Outcome variables section of the Methods section.*

2. Again, with this huge sample sizes, the p-value <0.05 is not clinically informative. The choice of predictors needs to be guided by clinical interpretability. Any discussion as to the clinically meaningful associations in the prediction model?

*As discussed above, the current model is intended primarily as a reference for investigators seeking to study outcomes in the CF population and to predict time to transplant or death. Extensive external validation would be required before introducing this nomogram into clinical care, as well as an updated analysis to reflect the benefits of HEMT.*

3. Table 3, why there are so many predictors with p of 0.0016? Were these from copy and paste errors? Instead of presenting raw-estimates and SEs, it would be better if authors use HR and its 95% CI. Also this applies to Table 2 where authors can report ORs and their CIs. Please do not include intercepts in each Table.

*As described in the Model Development section of Materials and Methods, “The Holm method was used to correct p-values for multiple testing [43].” Specifically, the R function p.adjust with method=holm was the correction methods for identifying p-values. Several of the predictors had initial p-values = 0.0000000000000002, i.e., the smallest value of p-value provided by R. When the Holm method was used to correct these p-values, the corrected p-values were 0.0016.*

*Thank you for your suggestions to report HR, OR, and 95% CI. We agree these make more sense to report since they are more easily interpreted. We have added estimated HR, OR, and 95% CI to Tables 2 and 3. We removed the intercept from Table 2. Since we felt reporting both 95% CI and p-values were redundant, we chose to remove the p-values from Tables 2 and 3.*

4. About missing data, the authors used the multiple imputation. What if authors use the completely observed data only, will the result be identical?

*Jakobsen et al. (2017) reported that complete case analysis should only be used if less than ~5% of observations are missing. In our study, the missingness ranged from 0.1% to 13%. Complete case analysis should also only be used if the missingness is unimportant, i.e., the missing observations are not impacting a specific portion of the sample (Jakobsen et al., 2017). In the case of our study population, it was possible that patients were lost to follow-up due to illness or being seen at a non-CF center hospital, so we did not feel comfortable assuming that the missing observations were not important. Jakobsen et al. (2017) additionally mentioned complete case analysis should only be used if the missing observations are only observed in the dependent and auxiliary variables, which was not the case with this study. Additionally, complete case analysis should only be used “if it is relatively certain that the data are MCAR,” which we did not believe was the case with our data (Jakobsen et al., 2017).*

5. As author mentioned, this cohort includes more whites than national-average. Any future accommodation for correcting this racial imbalance?

*Cystic Fibrosis (CF) is primarily observed in the white population. As noted in the CFF 2020 Patient Registry Annual Data Report, in 2020, 93.4% of the CFF Patient were white (https://www.cff.org/medical-professionals/patient-registry). This percent is very similar to the % of whites we saw in our death/transplant and alive populations, 95.6% and 93.5%, respectively, with (93.7% white overall). As noted in the Patients section of the Materials and Methods of our paper, “The CFFPR is estimated to include approximately 81-84% of persons with CF in the United States [41].” Thus, it should be roughly representative of the national CF population.*

*As a genetic disease, CF is not distributed evenly between all races. As noted by the CDC CF fact sheet, “The disease occurs mostly in whites whose ancestors came from northern Europe, although it affects all races and ethnic groups.” (*[*https://www.cdc.gov/scienceambassador/documents/cystic-fibrosis-fact-sheet.pdf*](https://www.cdc.gov/scienceambassador/documents/cystic-fibrosis-fact-sheet.pdf)*). With genetic testing and a better understanding of the disease, there has been an increase in the diagnosis of CF in non-white populations; however, in the United States the vast majority of patients are white, as reflected by the US Patent Registry.*

*Given the CF is naturally very prevalent of among whites, we do not have future plans to structure a CF cohort that matches the National US population diversity. Our sample is representative of the National CF population diversity.*

6. When reporting survival endpoint, it would be nicer if authors clarify the median follow-up time, frequency of censoring.

*Thank you for this helpful suggestion. We agree it is helpful information for the reader to know.*

*The median follow-up time was 8 years. We have added this information to the Patient Characteristics section of the Results section.*

*The frequency of censoring was 24,641 (82.6%) observations out of the total of 29,847 observations. This is reported in Table 1 and in the Patient Characteristics section of the Results section.*

6. PLOS authors have the option to publish the peer review history of their article ([what does this mean?](https://urldefense.com/v3/__https:/journals.plos.org/plosone/s/editorial-and-peer-review-process*loc-peer-review-history__;Iw!!BZ50a36bapWJ!uQ4kAL3oWT7Ewnzp0yEocSv8pKr7ekeASsvtfyeJeL6Mz0hnppQMujcAEUP9DKYdxCttNSWMcz3JJER8SghNlw$)). If published, this will include your full peer review and any attached files.

If you choose “no”, your identity will remain anonymous but your review may still be made public.

**Do you want your identity to be public for this peer review?** For information about this choice, including consent withdrawal, please see our [Privacy Policy](https://urldefense.com/v3/__https:/www.plos.org/privacy-policy__;!!BZ50a36bapWJ!uQ4kAL3oWT7Ewnzp0yEocSv8pKr7ekeASsvtfyeJeL6Mz0hnppQMujcAEUP9DKYdxCttNSWMcz3JJESmmfvT7g$).

Reviewer #1: No

Reviewer #2: No

[NOTE: If reviewer comments were submitted as an attachment file, they will be attached to this email and accessible via the submission site. Please log into your account, locate the manuscript record, and check for the action link "View Attachments". If this link does not appear, there are no attachment files.]

While revising your submission, please upload your figure files to the Preflight Analysis and Conversion Engine (PACE) digital diagnostic tool, [https://pacev2.apexcovantage.com/](https://urldefense.com/v3/__https:/pacev2.apexcovantage.com/__;!!BZ50a36bapWJ!uQ4kAL3oWT7Ewnzp0yEocSv8pKr7ekeASsvtfyeJeL6Mz0hnppQMujcAEUP9DKYdxCttNSWMcz3JJEQaCN0Z3g$). PACE helps ensure that figures meet PLOS requirements. To use PACE, you must first register as a user. Registration is free. Then, login and navigate to the UPLOAD tab, where you will find detailed instructions on how to use the tool. If you encounter any issues or have any questions when using PACE, please email PLOS at [figures@plos.org](mailto:figures@plos.org). Please note that Supporting Information files do not need this step.

References:

Austin, PC, and Steyerberg, EW. (2012). Interpreting the concordance statistic of a logistic regression model: relation to the variance and odds ratio of a continuous explanatory variable. *BMC Med Res Methodol* **12**, 82. <https://doi.org/10.1186/1471-2288-12-82>.

Facts about Cystic Fibrosis. CDC National Institutes of Health National Heart, Lung, and Blood Institute. <https://www.cdc.gov/scienceambassador/documents/cystic-fibrosis-fact-sheet.pdf>

Gottlieb, J. (2017). Lung allocation. *J. Thorac. Dis.*, Aug; 9(8) 2670-2674. doi: [10.21037/jtd.2017.07.83](https://doi.org/10.21037%2Fjtd.2017.07.83)

Harrell, F.E. rms: Regression Modeling Strategies, <https://cran.r-project.org/package=rms>, published 2024-03-11

Jakobsen, J.C., Gluud, C., Wetterslev, J., and Winkel, P. (2017). When and how multiple imputation should be used for handling missing data in randomized clinical trials – a practical guide with flowcharts. *BMC Med Res Methodol*, **17**, 162. <https://doi.org/10.1186/s12874-017-0442-1>

Ramos, K.J., Quon, B.S., Heltshe, S.L., Mayer-Hambeltt, N., Lease, E.D., Aitken, M.L., et al. (2017). Heterogeneity in Survival in Adult Patients with FEV1 < 30% of Predicted in the United States. *Chest*. 151(6): 1320-1328. https:/doi.org/10.1016/j.chest.2017.01.019.
