# Supplementary material for: Modeling cystic fibrosis patient prognosis: Nomograms to predict lung transplantation and survival prior to highly effective modular therapy
Source: PLoS One. 2024 Dec 5;19(12):e0292568. doi: 10.1371/journal.pone.0292568 (PMC11620394; doi:10.1371/journal.pone.0292568)
Supplement: Attachment — Submitted filename: Response to Reviewers .docx [file pone.0292568.s002.docx]

Reviewers' comments:

Reviewer's Responses to Questions

Comments to the Author

1. If the authors have adequately addressed your comments raised in a previous round of review and you feel that this manuscript is now acceptable for publication, you may indicate that here to bypass the “Comments to the Author” section, enter your conflict of interest statement in the “Confidential to Editor” section, and submit your "Accept" recommendation.

Reviewer #1: (No Response)

Reviewer #2: (No Response)

2. Is the manuscript technically sound, and do the data support the conclusions?

The manuscript must describe a technically sound piece of scientific research with data that supports the conclusions. Experiments must have been conducted rigorously, with appropriate controls, replication, and sample sizes. The conclusions must be drawn appropriately based on the data presented.

Reviewer #1: Yes

Reviewer #2: Partly

3. Has the statistical analysis been performed appropriately and rigorously?

Reviewer #1: Yes

Reviewer #2: No

4. Have the authors made all data underlying the findings in their manuscript fully available?

The [PLOS Data policy](https://urldefense.com/v3/__http:/www.plosone.org/static/policies.action*sharing__;Iw!!BZ50a36bapWJ!rpaFNILjeJTFl9bgrhsA3xzQMB8iAeTCYRvQ7clMWZ4RpY578ADnNQrgVDGP4UAxmqYyfVx69BFGmel6XuKdJQ$) requires authors to make all data underlying the findings described in their manuscript fully available without restriction, with rare exception (please refer to the Data Availability Statement in the manuscript PDF file). The data should be provided as part of the manuscript or its supporting information, or deposited to a public repository. For example, in addition to summary statistics, the data points behind means, medians and variance measures should be available. If there are restrictions on publicly sharing data—e.g. participant privacy or use of data from a third party—those must be specified.

Reviewer #1: No

Reviewer #2: Yes

5. Is the manuscript presented in an intelligible fashion and written in standard English?

PLOS ONE does not copyedit accepted manuscripts, so the language in submitted articles must be clear, correct, and unambiguous. Any typographical or grammatical errors should be corrected at revision, so please note any specific errors here.

Reviewer #1: Yes

Reviewer #2: Yes

6. Review Comments to the Author

Please use the space provided to explain your answers to the questions above. You may also include additional comments for the author, including concerns about dual publication, research ethics, or publication ethics. (Please upload your review as an attachment if it exceeds 20,000 characters)

Reviewer #1: Dear authors, thanks for taking into considerations my previous points.

Now I have only minor comments.

1.Patients who were already transplanted, were they excluded from the analysis? What is the prevalence of re-transplant in your dataset?

*Age of transplant and the indicator of lung transplant was based on a variable that indicated the year of first lung transplant so patients’ lives post-lung transplant and patients with a re-transplant were not considered. We have an explanation of this to the manuscript.*

2. LTx as a competitive event. I would have expected an analysis taking this into consideration, e.g. multi-state modelling, as done by this group: 10.2147/TCRM.S391476. Btw, this is an European cohort mostly pre-HEMT which may bring additional discussion with regards to time-varying covariates.

*Thank you for bringing this manuscript to our attention. We agree that LTx is a competitive event and understand why Gambazza et al. (2023) used multistate modeling to address the effect of oxygen therapy on disease progression and we added a reference to this manuscript in our paper given its relevance in being a pre-HEMT study. We disagree that we should do multistate modeling with our study because the aim of our study is different. Gambazza et al. (2023) was looking at disease progression, whereas our study is predicting probability of lung transplant/death. We agree these outcomes are related to disease progression; however, we were trying to assess whether and when a patient should be listed for lung transplant. The way Gambazza et al. (2023) set up their multistate model with alive with and without lung transplant does not make sense in the context of our study. While it is possible for patients to require a second lung transplant we instead focused time to first lung transplant/death, whichever came first.*

3. 6 minute walking test. Actually this is a variable available in the CFF Registry: its role has been debated in CF for LAS calculation but its variation cound have been useful (doi:10.1080/09638288.2022.2099588), since the existent relationship between exercise capacity and survival.

*Thank you for bringing this manuscript to our attention. Given this reference about the 6-minute walking test was not published prior to when we requested the data in 2016, we were unaware of its significance for LAS calculation. We instead chose to request predictors that had been well-referenced prior to 2016, as noted in the manuscript. Given the potential for this variable for this to be useful, it was included in our description of potentially informative predictors in the limitations section in our discussion in the manuscript. We had previously included another reference supporting the use of this variable, so we added this reference to further support its use.*

4. I'd suggest to adapt the manuscript following these recommendations.
- Steyerberg EW, Moons KGM, van der Windt DA, et al. Prognosis Research Strategy (PROGRESS) 3: Prognostic
Model Research. Plos Medicine. 2013; 10.

- Collins GS, Dhiman P, Ma J, Schlussel MM, Archer L, Van Calster B, Harrell FE Jr, Martin GP, Moons KGM, van Smeden M, Sperrin M, Bullock GS, Riley RD. Evaluation of clinical prediction models (part 1): from development to external validation. BMJ. 2024 Jan 8;384:e074819. doi: 10.1136/bmj-2023-074819. PMID: 38191193; PMCID: PMC10772854.

*Thank you for suggesting these manuscripts. We agree external validation and impact of using this model in clinical practice could provide further evidence of the benefits of using our models. We were unable to secure an external cohort in order to further validate our models and understand implementing them into clinical practice would require further study; however, we feel these models introduce a novel way of predicting probability and time to lung transplant that are more patient-specific than the current methods.*

- Collins GS, Reitsma JB, Altman DG, Moons KGM. Tr3ansparent reporting of a multivariable prediction model for individual prognosis or diagnosis (TRIPOD): the TRIPOD statement. BMJ 2015;350:g7594. doi:10.1136/bmj.g7594 18
- Moons KGM, Altman DG, Reitsma JB, et al. Transparent Reporting of a multivariable prediction model for Individual Prognosis or Diagnosis (TRIPOD): explanation and elaboration. Ann Intern Med 2015;162:W1-73. doi:10.7326/M14-0698

*Thank you for suggesting these manuscripts. We feel our manuscript thoroughly describes the items indicated as essential for reporting multivariable prediction models.*

Reviewer #2: I still have clarity concerns as below.

1. For the correlations, authors can generate the correlation plot to include 20x20 correlation matrix (corplot in R). Given that 20 predictors are not immense, authors can create a secondary table (similar to Table 1) that summarizes each predictor missing frequency and refer to it instead of listing them in the main text.

*We agree it’s helpful to visualize the correlation among the predictors; however, given the variety of predictors in this study, we do not feel it’ll be helpful in this context. We have continuous variables (e.g. fev1pp, height), binary categorical (e.g. race, P. aeruginosa positive), and ordinal variables (e.g. CFRD and F508 genotype); thus, used a variety of different correlation measures to appropriately address the different combinations of each pair of variables. To look at the association between pairs of continuous variables, we used Pearson correlation, whereas, with pairs of binary variables, we used tetrachoric correlation, and with pairs of polychoric correlation—and we used these 3 types of correlation appropriately when the pairs of predictors were not of the same time. We feel providing a correlation plot with all these different types of correlation would be confusing and unnecessary given we didn’t set out to assess associations between predictors, but used the associations to inform the combination of predictors we used in our prediction models (recall—the prediction models were the primary aim of this study.). We feel our level of detail with regard to correlation is sufficient.*

*We agree mentioning the amount of missingness observed among the predictors is necessary but we don’t feel it is necessary to add a table to describe this. Not all predictors had missing observations so we feel setting up the predictors with missing observations in a table would highlight these more so than the predictors without missing observations.*

2. Using AIC and p-value as guidance for inclusion of predictors will lead to overfitting given LARGE sample size. Authors need to compare the model with BIC or adjustedAIC were used for the model selection.

*We agree adding BIC will help justify our selection predictors and we have added it to our methods and analysis.*

3. The rationale behind using complete case analysis is see whether there is any possibility of non-random missing data. Again, given this large sample size, imputation or not-impute data analysis can lead to the similar final model.

*Thank you for your explanation of why you were interested in a complete case analysis. We agree that there is likely to be non-random missing data as we mentioned in our previous response: “In the case of our study population, it was possible that patients were lost to follow-up due to illness or being seen at a non-CF center hospital, so we did not feel comfortable assuming that the missing observations were not important.” We ran the complete case complete analysis and resulted in very similar results for both the logistic regression and the Cox regression models.*

4. Authors can briefly discuss the goodness-of-fit test results, Hosmer-Lemeshow or RMS omnibus test results for logistic models and Schonfeld test results for Cox regression.

*We agree a discussion of these will help more thoroughly describe our findings and have added a discussion of these to the manuscript.* *To assess the fit of the logistic model, we used a calibration plot of the predicted values against the observed values in order to visually observe and assess the fit, because the Hosmer-Lemeshow and RMS omnibus tests don’t allow that option.*

5. It is not clear how authors select the final model based upon this description "Full logistic and Cox regression models were fit including all independent variables of interest Significance of predictors (p ≤ 0.05),
Akaike information criterion (AIC), and concordance index were used to select the best subset of
predictors for the final models. The Holm method was used to correct p-values for multiple
testing [43]." Why authors considered multiple correction when multiple regression model was considered?

*Because the number of predictors in the final logistic and Cox models were 19 and 16, respectively, and given the sample size was large, we felt it was necessary to use the Holm method to minimize the potential for Type 1 error. With each step of model building, we checked the significance of the predictors, AIC, concordance index, and (upon this review) BIC, to determine if removing a predictor would improve the model, indicated by a meaningful decrease in AIC and BIC and a meaningful increase in concordance. The predictors were removed based on these and if their p-value was greater than 0.05.*

7. PLOS authors have the option to publish the peer review history of their article ([what does this mean?](https://urldefense.com/v3/__https:/journals.plos.org/plosone/s/editorial-and-peer-review-process*loc-peer-review-history__;Iw!!BZ50a36bapWJ!rpaFNILjeJTFl9bgrhsA3xzQMB8iAeTCYRvQ7clMWZ4RpY578ADnNQrgVDGP4UAxmqYyfVx69BFGmenBXnz-sw$)). If published, this will include your full peer review and any attached files.

No.

If you choose “no”, your identity will remain anonymous but your review may still be made public.


Do you want your identity to be public for this peer review? For information about this choice, including consent withdrawal, please see our [Privacy Policy](https://urldefense.com/v3/__https:/www.plos.org/privacy-policy__;!!BZ50a36bapWJ!rpaFNILjeJTFl9bgrhsA3xzQMB8iAeTCYRvQ7clMWZ4RpY578ADnNQrgVDGP4UAxmqYyfVx69BFGmek76SXFJQ$).

Reviewer #1: No

Reviewer #2: No
